# Transition regret and detransition: what went wrong?

**DOI:** 10.1192/j.eurpsy.2025.2295

**Published:** 2025-08-26

**Authors:** A. Cardoso, M. Mota

**Affiliations:** 1 Psychiatry Department, Unidade Local de Saúde de São João, Porto, Portugal

## Abstract

**Introduction:**

The phenomena of regret and detransition in individuals undergoing *gender*-*affirming treatments* raise significant medical and bioethical challenges for professionals working in this area.

**Objectives:**

This work aimed to gather the most current evidence regarding the approach to the issues of regret and detransition, characterizing the main factors involved and reflecting on possible prevention strategies.

**Methods:**

A literature review was conducted through research on PubMed, using the keywords “gender dysphoria,” “regret,” and “detransition”. Only articles in English were included. Additional bibliography was selected by consulting the references of the initially included articles.

**Results:**

Regret and detransition are distinct concepts. In fact, there can be regret without detransition and detransition without regret, with the narratives and experiences of these individuals being very diverse. Detransition may be motivated by external or internal factors, depending on whether transgender identity is preserved or lost. Originally thought to be rare, it has been challenging to assess the actual prevalence of these phenomena, whose increase is expected in the future. In an effort to counter this trend, the literature emphasizes the importance of a multidisciplinary and comprehensive approach to individuals with gender dysphoria, based on effective and assertive communication, ensuring responsible and informed decision-making. Regular follow-up combined with psychosocial support throughout the entire transition process is also crucial. Therapeutic approach should be individualized and integrated into a continuing care plan, grounded in an empathetic and non-judgmental attitude.

**Conclusions:**

Regret and detransition are not necessarily synonymous with medical error. Considering the complex spectrum of experiences involving these phenomena, a comprehensive approach that allows for an integrated view of each person and their needs is essential. Further research and development of guidelines regarding the approach and support for this group of individuals are needed.

**Disclosure of Interest:**

None Declared

